# Parents of Children With Neurodevelopmental Disorders: A Mixed Methods Approach to Understanding Quality of Life, Stress, and Perceived Social Support

**DOI:** 10.7759/cureus.37356

**Published:** 2023-04-10

**Authors:** Sarah Y Faden, Nisma Merdad, Yaser A Faden

**Affiliations:** 1 Psychology, Dar Al-Hekma University, Jeddah, SAU; 2 Psychology, Effat University, Jeddah, SAU; 3 Obstetrics and Gynaecology, King Saud Bin Abdulaziz University for Health Sciences College of Medicine, Jeddah, SAU

**Keywords:** perceived social support, parental stress, attention deficit hyperactivity disorder (adhd), quality of life (qol), autism spectrum disorder (asd)

## Abstract

Objective

This study aims to investigate the quality of life (QOL), parental stress, and perception of social support in parents of children with neurodevelopmental disorders (NDD) in Saudi Arabia.

Background

Studies have shown that parenting a child with NDD impacts the QOL, parental stress, and life satisfaction of parents. Those studies, however, assessed those factors separately as well as focusing on autism spectrum disorder (ASD). This study will use a mixed methods approach to gain a deeper understanding of those three factors as they related to parenting a child with NDD.

Method

Data about parental stress, QOL, and other related sociodemographic variables were collected from parents of children with NDD (N= 63). Next, semi-structured interviews were conducted with four of those parents to gain a deeper understanding of the parents’ QOL, parental stress, and perception of social support.

Results

An analysis of variance (ANOVA) test demonstrated that parents who had children with severe symptoms had poorer QOL and higher levels of parental stress compared to parents who had children who had moderate and mild symptoms. In addition, parents who had children with ASD had poorer QOL compared to all of the other disorders. There was no statistically significant difference in QOL and parental stress between mothers and fathers. The thematic analysis highlighted that the most significant challenges they face are financial, familial, and well-being worries.

Conclusion

In conclusion, this study shows that parents of children with NDD exhibited higher levels of parental stress and lower levels of QOL depending on the diagnosis and intensity of the child’s symptoms. In addition, the interviews highlighted some key challenges that the parents felt affected their QOL and stress levels, as well as their views on family, friends, and community social support.

Implications

This study can help in developing or improving supportive programs and interventions for parents with children with NDD to enhance their QOL, reduce parental stress, and create a better social support system.

## Introduction

Parenting a child with a neurodevelopmental disorder (NDD) is a challenging journey impacting many areas of the parent’s life. According to the Diagnostic and Statistical Manual of Mental Disorders, 5th edition (DSM-5), “NDDs are characterized by developmental deficits that produce impairments of personal, social, academic, or occupational functioning” [1]. Thus, NDDs may include intellectual disabilities, communication disorders, autism spectrum disorder (ASD), attention-deficit/hyperactivity disorder (ADHD), specific learning disorders, and motor disorders. A parent learning that their child is diagnosed with an NDD begins a journey often filled with financial, social, emotional, and psychological challenges that impact their quality of life (QOL), stress levels, and perceived social support. The World Health Organization (WHO) defines QOL “as an individual's perception of their position in life in the context of the culture and value systems in which they live and in relation to their goals, expectations, standards, and concerns” [2].

Research has shown that having a child with an NDD negatively impacts the parents’ QOL, and parents of children with ASD, in particular, report a poorer QOL than parents of typically developed children [3]. According to another more recent study, caregivers of children with NDD reported experiencing increased tiredness and feelings of unwellness [4] as well as high levels of parental stress, defined as the mental or emotional strain or pressure that an individual experiences due to being a parent [5]. To help them manage these stressors, they may desperately need social support, and so how much parents perceive the quality and degree of psychological and overall support provided by family members, friends, and others, (i.e., their perceived social support) takes on added importance [6].

Previous studies have shown that having a child with an NDD impacts many aspects of the parent’s life. A study conducted in Arar, Saudi Arabia, involving 84 parents of children with ASD found that 63.1% had impaired QOL. Moreover, parents of children having severe ASD were nearly three times more likely to suffer from poor QOL than those with typically developed children [7]. Another study conducted in Saudi Arabia to evaluate families’ challenges in raising a child with ASD showed that such families suffer from many psychological, social, emotional, and financial challenges [8]. Still, another study conducted in Bangladesh involving 904 caregivers of children diagnosed with NDDs sought to explore parental stress, including potential contributing factors related to the children’s life events [9]. It found parental stress to be higher among caregivers who were female, less well-educated, and unemployed. Moreover, parental stress was found to be highly and positively associated with the child’s age and the seriousness of the NDD. Moreover, a study conducted on 104 mothers of children with NDDs in Pakistan to explore the relationship between the social support they received, their levels of stress, and their overall life satisfaction found the impact of social support on the mothers’ life satisfaction to be significant [10].

Even though many studies conducted prior to ours examined parental QOL, levels of stress experienced, and perceived social support, their analyses failed to incorporate all three of these factors, and none were situated in Saudi Arabia. Moreover, whereas the focus of most was ASD specifically, our research incorporated parents of children with varied types of NDDs. Also differing was our approach, a mixed-methods one intended to provide a deeper understanding of parents’ QOL, parental stress, and perceived level of social support. Understanding the experiences, struggles, and challenges parents of NDD children face is necessary to develop or improve support programs and interventions for these parents so as to improve their QOL, reduce their parental stress, and so raise their perceived levels of social support. Therefore, the aim of this study was to investigate the QOL, parental stress, and perceptions of social support of parents of children with NDDs in Saudi Arabia.

Research question

Does the care of a child having an NDD impact their parents’ QOL, levels of parental stress, and perceived social support level?

## Materials and methods

This research employed a mixed methods approach, i.e., one using quantitative and qualitative techniques, and its subjects were NDD-children parents in Saudi Arabia. First, quantitative measures of parental QOL and stress levels were collected using a cross-sectional survey, and then semi-structured interviews were employed to obtain a deeper understanding of these parents’ experiences and, in addition, their perceptions of the social support they received. Dar Al-Hekma University reviewed and approved the research study, which was conducted in March 2022. A pilot study was performed with seven participants before the questionnaire was made available to the full study’s participants.

Participants

Table 1 and Table 2 show the parents’ and child’s demographic information.

**Table 1:** Parents' Demographic Information ASD: autism spectrum disorder ADHD: attention deficit hyperactivity disorder

					Relationship with child				
					Mother		Father		
Variable					Frequency n	Percentage %	Frequency n	Percentage %	Chi-Square
Age of parent									0.143
	25-34				8	29.6%	1	6.3%	
	35-49				14	51.9%	12	75.0%	
	50-64				5	18.5%	2	12.5%	
	65+				0	0.0%	1	6.3%	
City									0.298
	Jeddah				20	74.1%	14	87.5%	
	Riyadh				3	11.1%	0	0.0%	
	Makkah				1	3.7%	0	0.0%	
	Madinah				1	3.7%	2	12.5%	
	Others				2	7.4%	0	0.0%	
Nationality									0.537
	Saudi				25	92.6%	16	100%	
	Syrian				1	3.7%	0	0.0%	
	Others				1	3.7%	0	0.0%	
Marital status									0.578
	Married				25	92.6%	14	87.5%	
	Divorced				2	7.4%	2	12.5%	
Relationship with partner									0.757
	Excellent				13	52.0%	8	57.1%	
	Moderate				12	48.0%	6	42.9%	
	Mild				0	0.0%	0	0.0%	
Level of education									0.131
	Did not graduate high school				1	3.7%	0	0.0%	
	High school graduate				1	3.7%	1	6.3%	
	Bachelor’s degree				17	63.0%	9	56.3%	
	Master’s degree				7	25.9%	1	6.3%	
	Doctoral degree				1	3.7%	4	25.0%	
	Others				0	0.0%	1	6.3%	
Employment status									0.102
	Full-time employment				9	33.3%	12	75.0%	
	Part-time employment				3	11.1%	0	0.0%	
	Unemployed/seeking work				2	7.4%	1	6.3%	
	Unemployed/not seeking work				8	29.6%	1	6.3%	
	Student				2	7.4%	0	0.0%	
	Retired				3	11.1%	2	12.5%	
Monthly household income									0.164
	Less than 5,000 SR				1	3.7%	1	6.3%	
	5,001 to 10,000 SR				10	37.0%	1	6.3%	
	10,001 to 15,000 SR				3	11.1%	3	18.8%	
	15,001 to 20,000 SR				4	14.8%	6	37.5%	
	Over 20,001 SR				9	33.3%	5	31.3%	
Child’s diagnosis									0.821
	Communication disorders				2	7.4%	1	6.3%	
	ASD				13	48.1%	6	37.5%	
	ADHD				10	37.0%	6	37.5%	
	Specific learning disorders				1	3.7%	1	6.3%	
	Others				1	3.7%	2	12.5%	

**Table 2:** Child’s Demographic Information ASD: autism spectrum disorder ADHD: attention deficit hyperactivity disorder

					Child’s gender						
					Female				Male		
Variable					Frequency n			Percentage %	Frequency n	Percentage %	Chi-Square
Age of child											0.357
	0-4				2			20.0%	5	15.2%	
	4-8				2			20.0%	11	33.3%	
	8-12				1			10.0%	9	27.3%	
	12-16				5			50.0%	8	24.2%	
Number of siblings											0.223
	0-1				2			20.0%	11	33.3%	
	2-3				4			40.0%	16	48.5%	
	4-5				3			30.0%	6	18.2%	
	6-7				1			10.0%	0	0.0%	
Child’s order											0.433
	1-2				4			40.0%	22	66.7%	
	3-4				3			30.0%	7	21.2%	
	5-6				2			20.0%	3	9.1%	
	7-8				1			10.0%	1	3.0%	
Diagnosis of child											0.743
	Communication disorders				1			10.0%	2	6.1%	
	ASD				4			40.0%	15	45.5%	
	ADHD				4			40.0%	12	36.4%	
	Specific learning disorders				1			10.0%	1	3.0%	
	Others				0			0.0%	3	9.1%	
Age when diagnosed											0.195
	0-3				5			50.0%	16	48.5%	
	3-6				3			30.0%	16	48.5%	
	6-9				1			10.0%	1	3.0%	
	9-12				1			10.0%	0	0.0%	
Intensity of child’s symptoms											0.995
	Mild				4			40.0%	13	39.4%	
	Moderate				5			50.0%	17	51.5%	
	Severe				1			10.0%	3	9.1%	
Child’s medications											0.897
	No				8			80.0%	27	81.8%	
	Yes				2			20.0%	6	18.2%	

For the qualitative part of the research, interviews were conducted with four participants (two fathers and two mothers) recruited based on the information collected from the survey and ranging in age from 25 to 40 years old. Table 3 presents the participants’ demographic data.

**Table 3:** Demographic Information of Interview Participants ASD: autism spectrum disorder ADHD: attention deficit hyperactivity disorder

Participants	Age in years	Gender	Marital status	Diagnosis of child	Age of child
1	25	Female	Married	ASD	2.5
2	32	Female	Married	ASD	4
3	36	Male	Married	ASD	10
4	40	Male	Divorced	ASD and ADHD	9

Study instruments

Questionnaire

An online survey instrument was designed to obtain data on parents' QOL and parental stress levels, and it was assembled in an electronic format using the Google Forms application. The questionnaire had four sections: (1) parent demographic information, (2) child demographic information, (3) the World Health Organization Quality of Life Brief Version (WHOQOL-BREF) questionnaire, and (4) the Parental Stress Scale (PSS). The first two sections contained questions designed to collect parent and NDD child demographic information.

Concluding the survey was an invitation to participate voluntarily in a follow-up interview, including a request for any participant willing to do so to enter their contact information (i.e., name, email, and phone number). Most of the questions on the Google form were designated mandatory, thereby eliminating the possibility of incomplete or missing data. The questionnaire took approximately 15 minutes to complete, and all data so collected were analyzed using the Statistical Package for the Social Sciences (SPSS) software, version 28.0.1.1 (IBM SPSS Statistics for Windows, Armonk, NY).

WHOQOL-BREF Questionnaire

A well-known tool designed by the World Health Organization (WHO), the WHOQOL-BREF survey instrument collects information needed to assess various subjective elements related to a respondent’s QOL [11]. The instrument includes 26 questions and covers four different domains of QOL: physical health (seven items), psychological health (six items), social relationships (three items), and environmental health (eight items). It also contains general health and QOL items. The possible responses to each item are designated on a 5-point Likert scale ranging from “very dissatisfied” (1) to “very satisfied” (5); from “not at all” (1) to “an extreme amount” (5); or from “never” (1) to “always” (5). The WHOQOL-BREF has been translated into many languages, including Arabic, as was the version employed in this research. With a Cronbach's alpha (α) of .926, the questionnaire's reliability was found to be excellent.

Parental Stress Scale (PSS)

An instrument developed in 1995 to assess the level of stress experienced by parents [12], the PSS focuses on both the positive and negative aspects of being a parent. The tool consists of 18 questions rated on a 5-point Likert scale with responses ranging from “strongly disagree” (1) to “strongly agree” (5). A professional translator translated the PSS into Arabic using a forward-backward procedure, and the scale’s reliability was satisfactory, with a Cronbach’s alpha (α) of .853.

Interview

Following the administration of the survey instrument, an interview schedule was created so as to capture as much information as possible about the participants' experiences. The semi-structured interviews were conducted one-on-one via phone. Although the parents interviewed were asked a total of seven open-ended questions designed to not only further explore their QOL in different areas and the levels of parental stress they experienced but also their perceptions of the level and quality of social support provided by family, friends, and community. Each interview lasted between 16 and 48 minutes and was conducted in Arabic, after which a transcript was created, translated into English by a professional translator, and then analyzed using Taguette online software (Rémi Rampin, Vicky Rampin, and Sarah DeMott; https://www.taguette.org/).

Ethical considerations

After gaining the approval of the Health and Behavior Science Education (HSBE) school research committee (Spring 21/22, Meeting #2: approval granted for undergraduate student research), the survey instrument was distributed to those parents willing to participate. Before completing the survey, participants were provided with a consent form that contained information about the research being conducted, including its objectives and purpose and ethical considerations regarding their participation and responses. All were informed that their responses would remain anonymous, that their participation in the survey was completely voluntary, and that no identifying information would be collected, except for those participants willing to also participate in an interview following the survey. After the survey responses were collected, they were placed in a password-protected file on the researcher’s laptop where no one but the researcher could access them.

After being informed of the interviews’ objectives and purpose, those parents willing to be interviewed were informed that their interviews would be audio recorded, that these recordings would be erased following completion of the study, that no identifying information disclosed during the interviews would be disclosed to others, and that they could cease participation at any point during the interview if they so desired. They were then asked to sign a consent form.

Power analysis

A power analysis was conducted based on a previous study conducted by Miranda et al. [13], which also involved parents of children with ASD. In this study, the correlation between the child’s ASD symptoms and the results obtained from the administration of the Strengths and Difficulties Questionnaire (SDQ-Tot) was 0.39. In our study, Type I error was set at .05, and Type II error at .20. The minimal sample size needed for validity was therefore found to be 49 respondents. Also, approximately four participants were needed for the interview.

## Results

Statistical analysis

Preliminary Analysis

Correlations between the study variables were calculated and are reported in Table 4 below.

**Table 4:** Pearson Correlations for the Study Variables QOL: quality of life SD: standard deviation

		Mean	SD	1	2	3		4	5	6	7	8	9	10	11	12	13
1	QOL	88.465	16.377	1.000													
2	Parental stress	48.047	11.397	-0.464**	1.000												
3	Relationship with child	1.370	0.489	0.058	-0.106	1.000											
4	Age of parent	3.000	0.690	0.082	0.006	0.212		1.000									
5	Marital status	1.190	0.588	-0.261	-0.158	0.085		0.000	1.000								
6	Education	5.560	14.613	-0.144	-0.005	0.210		0.019	0.481**	1.000							
7	Employment	2.600	1.841	0.025	0.049	-0.282		0.319*	-0.062	0.021	1.000						
8	Household income	3.530	1.316	0.371*	-0.153	0.164		0.315*	-0.255	-0.282	-0.068	1.000					
9	Age of child	2.670	1.085	0.187	-0.201	0.234		0.604**	0.172	0.055	0.232	0.258	1.000				
10	Child’s gender	1.770	0.427	-0.069	-0.149	0.310*		-0.161	0.176	0.086	-0.271	-0.070	-0.116	1.000			
11	Diagnosis	10.090	24.646	0.014	-0.205	0.169		0.270	0.229	0.577**	0.106	-0.180	0.088	0.149	1.000		
12	Intensity	1.700	0.638	-0.290	0.510**	-0.089		0.000	0.027	-0.168	-0.125	-0.030	0.061	0.002	-0.022	1.000	
13	Number of siblings	1.950	0.785	0.122	0.136	0.046		0.571**	-0.187	-0.176	0.135	0.163	0.345*	-0.246	0.022	-0.029	1.000
	**. Correlation is significant at the 0.01 level (2-tailed). *. Correlation is significant at the 0.05 level (2-tailed).																

Outcomes of severity and type of diagnosis

One-way analysis of variance (ANOVA) was employed to identify statistically significant group differences between QOL and parental stress of children’s diagnosis with respect to the intensity of symptoms (mild, moderate, and severe). Post-hoc analyses were conducted to explore significant differences between specific groups. Since the children of the majority of survey respondents had ASD and ADHD and the number of parents having children with communication disorders, specific learning disorders, or other disorders was insufficient, these parents' data were combined into a single category denoted “others.”

QOL

An ANOVA showed that the QOL with respect to the intensity of the children’s symptoms was significant with p = .041 (see Table 5). Post-hoc analyses indicated a statistically significant difference in QOL for mild and severe symptoms (p = .036) but no significant differences for mild and moderate (p = .936) and moderate and severe ones (p = .051). Moreover, an ANOVA showed the QOL differentiated with respect to children’s diagnoses to also be statistically significant (p < .001).

**Table 5:** ANOVA results test for the quality of life * Indicates a significance level < .05 ANOVA: analysis of variance QOL: quality of life ASD: autism spectrum disorder ADHD: attention deficit hyperactivity disorder

			QOL				p-value
Intensity of child’s symptoms			n	Mean	SD	95% CI	
	Mild		17	91.4	11.9	85.3-97.5	0.041*
	Moderate		22	89.7	16.1	82.5-96.8	
	Severe		04	69.3	25.1	29.3-109.2	
Diagnosis of child							
	ASD		19	78.6	16.6	70.6-86.6	< 0.001*
	ADHD		16	98.3	11.8	91.99-104.5	
	Other		08	92.4	10.2	83.8-100.9	

Parental stress

An ANOVA showed that the level of parental stress with respect to the intensity of the child’s symptoms was significant (p = .002) (see Table 6). Post-hoc analyses indicated a statistically significant difference between parental stress levels for mild and moderate symptoms (p = .005) and mild and severe ones (p = .015). However, there was no significant difference in parental stress for moderate and severe symptoms (p = .575). Moreover, an ANOVA showed that parental stress with respect to the seriousness of children’s diagnoses was significant (p = .001).

**Table 6:** ANOVA test results for parental stress * Indicates a significance at below .05 ANOVA: analysis of variance ASD: autism spectrum disorder ADHD: attention deficit hyperactivity disorder

			Parental stress				p-value
Intensity of child’s symptoms			n	Mean	SD	95% CI	
	Mild		17	41.1	7.7	37.1-45.02	0.002*
	Moderate		22	51.8	11.9	46.5-57.1	
	Severe		4	57.3	11.9	51.1-63.4	
Diagnosis of child							
	ASD		19	54.7	9.3	50.2-59.2	0.001*
	ADHD		16	43.9	11.6	37.5-49.9	
	Others		8	41.0	7.1	35.1-46.9	

Outcomes of differences between parents (mothers and fathers)

Also performed were t-tests to measure the differences between mothers’ and fathers’ QOL and reported levels of parental stress. No statistically significant difference in QOL between mothers (M = 87.7, SD = 16.4) and fathers (M = 89.7, SD = 16.8) of children with NDD was found (p = .7). Moreover, no statistically significant difference in parental stress levels between mothers (M = 48.96, SD = 11.5) and fathers (M = 46.5, SD = 11.5) of children with NDD was found (p = .5).

Thus, parental stress and QOL were found to be strongly linked to the intensity of the child's symptoms and type of disorder. Although this indicates the presence of differences, their causes were unknown, prompting the study interviews, whose intent was to reveal the origins of the parental stress and the events that affected both it and QOL.

Semi-structured interviews

A thematic analysis of the transcripts of the interviews was conducted using Taguette, and Table 7 presents the superordinate, master, and emergent themes discovered.

**Table 7:** Emergent themes generated from semi-structured interviews ASD: autism spectrum disorder ABA: applied behavior analysis BCBA: board-certified behavior analyst NDD: neurodevelopmental disorder

Superordinate themes	Master themes		Emergent themes	Participants’ comments
Challenges	Financial		Therapy (ABA, speech sessions, and assessments), private schools, transportation, and more expensive than abroad	Most mentioned how expensive ABA, speech, and assessment therapy sessions were, especially good ones (Participants 1 and 3). Some cited the cost of schools, particularly private ones (Participants 3 and 4).
	Family		Lack of acceptance and support, and hiding from family members	Participant 2 stated, “There is no support from the family; they consider the mother responsible for everything.” Some parents (Participant 1) sought to hide their child’s condition from other family members, and this effort constituted still another challenge.
	Well-being		Need for support and acceptance, caring and dealing with the child, internal struggles, pretending to be emotionally stable, heavy burden, and no social support	Participants reported experiencing internal struggles, feelings associated with carrying a heavy burden, “dealing with … a great responsibility you never expected” (Participant 2), and psychological pressure (Participant 1). Participant 1 also reported feeling the need to pretend to be emotionally stable in spite of these feelings: “A challenge I face is how I show myself in front of others that I am good and well when truly I am not.”
	Quality of life		Lack of social awareness, awkwardness, and lack of empathy from society, integrating with people, and not going out for entertainment	Participant 2 mentioned a “high lack of awareness about children with NDDs” and a lack of empathy from society in general: “Communities are full of problems, and society is full of competition, hatred, and malice among people; in other words, they are negative from the start.” Due to this lack of awareness, “when we go outside the house,” Participant 4 stated, “my child’s movements cause us some awkwardness, and people do not understand this.”
	Parental stress		Fear when upbringing the child, worry about the child’s future, responsibility, separation anxiety, and unknown source of stress	“Having a child with ASD is a great responsibility; I feel afraid when I take care of him, and I wonder if I am dealing with him properly or not,” Participant 2 stated, adding, “I am afraid that anything will happen to him in the future.” Participant 3 also reported worry about their child’s future, particularly when feeling stressed.
Support system	Family support		Babysitting, psychological and moral support, providing resources, and equating treatment between the child and other children	Most of that provided by family consisted of “babysitting” when needed (Participants 1 and 3), “house chores,” and other “sources” of aid (Participant 1).
	Friends support		Positive feelings, psychological support, ask and play with the child, and accommodating the child's situation	“They help me psychologically, and they entertain me, regardless of whether I tell them that I am upset or not,” said Participant 1. Describing their friends, Participant 4 stated, “They always ask about my son, and we go out together and play with him.” In addition, this participant felt that these friends were “accommodating his situation”
	Community support	Accessible and helpful	Developmental pediatricians and psychiatrists, specialists with BCBA and psychologists, ABA sessions, and books	Participant 1 stated, “I registered my child in a nursery with individual and collective behavioral modification sessions. In addition, we go to Applied Behavior Analysis (ABA) therapy sessions in the evening twice a week, and I feel that this is very useful to my child.” Participant 3 reported, “The experts we see are the ones who provide us with the support we need; for example, there is a psychiatrist who does not mind if we call her at any time we need.”
		Inaccessible	No place that provides all services needed, no guide for services available, and activities for children with ASD	Some mentioned that no one place provided all the services needed, and, Participant 1 added, “They would be more efficient if they were given in one place.” Participant 1 also cited their country’s lack of a guide listing all services available to NDD parents: “If there were a clear guide to everything, to the services and things provided, this would help me a lot.” Another concern was that “[t]here are no activities available for the children and their parents where they can meet with others in similar situations and enjoy their time” (Participant 4).
		Accessible but unhelpful	Schools centers and services, insufficient availability of specialists, and financial support from governmental sectors	Participant 1 stated, “A service we seek is for our child to get the support and resources of the Ministry of Human Resources and Social Development, but even if it does happen, still, the financial support is considered low based on the expensive services we require.” Participant 3 also stated, “[T]here is a lack of excellent services for cases like my child.” Participant 3 cited a “lack of good centers or schools” and stated that the “schools are worse than the centers. Worse, in terms of services.”

## Discussion

Questionnaire

The aim of the study was to investigate the QOL, parental stress levels, and perceptions related to the social support of parents of children having NDDs in Saudi Arabia. The study results showed that parents of children exhibiting severe symptoms of NDDs scored lower in QOL and higher in parental stress than did parents of children who exhibited moderate and mild symptoms. In addition, parents of children with ASD displayed lower scores of QOL compared to those whose children had ADHD and other types of disabilities. Moreover, the mothers and fathers exhibited no statistically significant differences in QOL and parental stress levels.

Interviews

The challenges faced by the parents and their perceptions of their support systems were the primary topics of discussion in the interviews. Taguette generated superordinate themes from the transcribed interviews of the study participants (see Table 7) from which it then generated emergent themes based on the details given upon discussion of the master themes.

Challenges

In general, the parents reported experiencing financial, familial, and well-being challenges, many based on their perceptions of their QOL and of the parental stress they experienced as a direct consequence of having a child with an NDD. All participants mentioned experiencing financial challenges, and most mentioned how expensive ABA, speech, and assessment therapy sessions were, especially good ones (Participants 1 and 3). Some cited the cost of schools, particularly private ones (Participants 3 and 4), and the difficulty they experienced in arranging their children’s transportation to school and therapy sessions (Participant 2).

Some participants reported familial challenges attributable to other family members’ lack of acceptance and support. For instance, Participant 2 stated, “*There is no support from the family; they consider the mother responsible for everything.*” Some parents (Participant 1) sought to hide their child’s condition from other family members, and this effort constituted still another challenge.

When asked about challenges that influenced their well-being, participants reported experiencing internal struggles, feelings associated with carrying a heavy burden, “*dealing with … a great responsibility you never expected*” (Participant 2), and psychological pressure (Participant 1). Participant 1 also reported feeling the need to pretend to be emotionally stable in spite of these feelings: “*A challenge I face is how I show myself in front of others that I am good and well when truly I am not.*” Thus, caring for and dealing with the child and his or her needs, the need for support and acceptance, and the lack of social support played a significant role in lessening these parents' sense of well-being.

Multiple factors associated with their children’s conditions negatively affected their QOL, many parents reported. For instance, Participant 2 mentioned a “*high lack of awareness about children with NDDs*” and a lack of empathy from society in general: “*Communities are full of problems, and society is full of competition, hatred, and malice among people; in other words, they are negative from the start.*” Due to this lack of awareness, “w*hen we go outside the house,*” Participant 4 stated, “*my child’s movements cause us some awkwardness, and people do not understand this*.” Socializing with others was a challenge for some parents, adding to their negative emotions. Also, because the parents' primary focus was their children, they failed to set aside time for their own entertainment and relaxation (Participant 1).

The parents also highlighted specific factors that heightened their levels of parental stress, including questioning their adequacy to best care for their children; “*Having a child with ASD is a great responsibility; I feel afraid when I take care of him, and I wonder if I am dealing with him properly or not,*” Participant 2 stated, adding, “*I am afraid that anything will happen to him in the future.*” Participant 3 also reported worry about their child’s future, particularly when feeling stressed. Another source of parental stress was separation anxiety. Parent 4 stated, *“There can be some pressure when my child is away from me in school, and I have not seen him, but if he is beside me, there is no stress.*”

As can be seen from the participants’ responses, QOL, parental stress, and parental lack of well-being are all linked to a variety of problems and circumstances associated with their children’s condition. Each participant faced difficulties as a consequence of this and of their environment.

Support system

In spite of the QOL and stress issues they experienced, the majority of participants reported receiving adequate and varied forms of support from family, friends, and the community. Most of that provided by family consisted of “*babysitting*” when needed (Participants 1 and 3), “*house chores,*” and other “*sources*” of aid (Participant 1). In addition, Participant 4 described family members’ failure to distinguish between their NDD child and their other, presumably non-NDD children as extremely gratifying; “*Their support is by treating all our children equally, not making him feel different from the rest; this makes me feel happy.*” Finally, families provided psychological/moral support: “*It is nice to feel that there are people around you, providing support beside you, and caring about you; that makes me feel comfortable*” (Participant 1).

Some participants reported receiving support from friends also. “*They help me psychologically, and they entertain me, regardless of whether I tell them that I am upset or not*,” said Participant 1. Describing their friends, Participant 4 stated, “*They always ask about my son, and we go out together and play with him*.” In addition, this participant felt that these friends were “accommodating his situation”: “*I feel like they are doing their best and coming down to my son's level of thinking and making sure he understands them, and they understand him.*”

Community support of the parents was divided into three subcategories based on accessibility and utilization: Accessible and Helpful, Inaccessible and Accessible but Unhelpful. Participant 4 reported that some doctors’ help was “*beneficial*,” and the accessible and helpful community support provided included developmental pediatricians, psychiatrists, ABA specialists, and psychologists offering different services. Participant 1 stated, “*I registered my child in a nursery with individual and collective behavioral modification sessions. In addition, we go to Applied Behavior Analysis (ABA) therapy sessions in the evening twice a week, and I feel that this is very useful to my child*.” Participant 3 reported, “*The experts we see are the ones who provide us with the support we need; for example, there is a psychiatrist who does not mind if we call her at any time we need.*”

However helpful, the parents communicated that the accessibility of community support could be improved. Some mentioned that no one place provided all the services needed, and, Participant 1 added, “*They would be more efficient if they were given in one place.*” Participant 1 also cited their country’s lack of a guide listing all services available to NDD parents: “*If there were a clear guide to everything, to the services and things provided, this would help me a lot*.” Another concern was that “*[t]here are no activities available for the children and their parents where they can meet with others in similar situations and enjoy their time*” (Participant 4).

Several participants also raised concerns about the unavailability of specialists and governmental funds directed to the needs of the children and their parents. Participant 1 stated, “*A service we seek is for our child to get the support and resources of the Ministry of Human Resources and Social Development, but even if it does happen, still, the financial support is considered low based on the expensive services we require.*” Participant 3 also stated, “*[T]here is a lack of excellent services for cases like my child.*”

Participant 3 cited a “*lack of good centers or schools*” and stated that the “*schools are worse than the centers. Worse, in terms of services.*” Participant 4 also cited schools as lacking: “*[T]he real problem lies in schools due to lack of appropriate services.*” However, the parents stated that the centers did not provide all the services they felt were needed or lacked sufficient resources to adequately provide the care to all that needed it. Participant 3 stated, “*I first looked at the best centers, specialists, and consultants. But in the end, I did not get the result I wanted, maybe because of the excess pressure on them.*”

Parents of children with NDDs appear to require support from a variety of sources in order to feel reassured and to experience reduced stress levels. Some participants were pleased with the level of support received, whereas others were dissatisfied due to what they perceived as a lack of support.

Several previous studies have investigated the QOL, parental stress level, and perceived social support of parents of children with NDDs, resulting in findings that both agreed and disagreed with those of our study. Whereas one such study concluded that caregivers of children with autism had reduced QOL regardless of the characteristics of the children’s symptoms [7], our research found that QOL scores of parents of children with NDDs were strongly and negatively associated with the intensity of the children’s symptoms. Still, another study found parental stress to be higher among female and less educated caregivers and to be associated with the diagnosis of NDD and the increasing age of the child [9]. Similarly, our research findings also found parental stress levels to be highly and positively associated with the severity of the child’s diagnosis. However, unlike the study of Haque et al., our study identified no difference in the parental stress experienced by male and female caregivers. Another study found social support to have a significant impact on the life satisfaction of mothers raising children with NDDs [10] a finding that matched our research, in which the interviews indicated that parents' QOL and parental stress levels were associated with the parents’ support system. Lastly, a study conducted in Saudi Arabia found that families of children having autism experienced many psychological, social, emotional, and financial challenges [8] and our research identified similar challenges experienced by parents of children with NDDs.

Limitations of study

The current study had some limitations. First, the sample size was less than that the initial power analysis recommended (43 vs. 49). Second, the majority of the participants had children with ASD and ADHD with only a small number exhibiting other disorders. Finally, the use of snowball sampling to select participants may have resulted in certain biases, as it resulted in most participants being from Jeddah. Thus, for future studies, participants with children having a greater variety of disorders should be included. Moreover, further research is also recommended to identify other variables influencing parents' lives, and a focus of future studies should be developing appropriate coping strategies for parents.

## Conclusions

In conclusion, our study showed that parents of children with NDDs experience significantly lowered QOL as well as increased parental stress levels due to dealing with the various challenges their children's conditions entail. As this study showed, the levels of parental stress and the degradation of QOL these parents experienced depended on the severity of their children’s diagnoses and the intensity of their symptoms. Furthermore, these do not differ between mothers and fathers. In addition, as revealed by the participants, the most significant challenges they face are financial, familial, and support related, all of which affect their sense of well-being, their QOL, and their levels of parental stress. The research findings thus emphasize the need to direct more attention to parents having children with NDDs so as to understand their perspectives and the adversities they experience with the aim of establishing and improving interventions and programs that will enhance their QOL, reduce their parental stress, and improve their perceptions of social support.
